# Endemic Scrub Typhus–like Illness, Chile

**DOI:** 10.3201/eid1709.100960

**Published:** 2011-09

**Authors:** M. Elvira Balcells, Ricardo Rabagliati, Patricia García, Helena Poggi, David Oddó, Marcela Concha, Katia Abarca, Ju Jiang, Daryl J. Kelly, Allen L. Richards, Paul A. Fuerst

**Affiliations:** Author affiliations: School of Medicine, Pontificia Universidad Católica de Chile, Santiago, Chile (M.E. Bacells, R. Rabagliati, P. García, H. Poggi, D. Oddó, M. Concha, K. Abarca);; Naval Medical Research Center, Silver Spring, Maryland, USA (J. Jiang, A.L. Richards);; The Ohio State University, Columbus, Ohio, USA (D.J. Kelly, P.A. Fuerst)

**Keywords:** scrub typhus, scrub typhus–like illness, Orientia tsutsugamushi, rickettsia, Chile, 16S rRNA, parasites, research

## Abstract

We report a case of scrub typhus in a 54-year-old man who was bitten by several terrestrial leeches during a trip to Chiloé Island in southern Chile in 2006. A molecular sample, identified as related to *Orientia tsutsugamushi* based on the sequence of the16S rRNA gene*,* was obtained from a biopsy specimen of the eschar on the patient’s leg. Serologic analysis showed immunoglobulin G conversion against *O. tsutsugamushi* whole cell antigen. This case and its associated molecular analyses suggest that an *Orientia*-like agent is present in the Western Hemisphere that can produce scrub typhus–like illness. The molecular analysis suggests that the infectious agent is closely related, although not identical, to members of the *Orientia* sp. from Asia.

## Abstract 2

TOC Summary: Rickettsiae closely related to the scrub typhus agent are present in the Western Hemisphere.

The primary hosts for *Rickettsia* species are arthropods that can also act as disease vectors for humans and other vertebrates. Ticks are vectors for most rickettsioses caused by spotted fever group rickettsiae. Alternative vectors for rickettsiae are well known, including fleas as vectors for murine typhus (*R. typhi*) and flea-borne spotted fever (*R. felis*)*,* mites as vectors of rickettsialpox (*R. akari*), and scrub typhus (*Orientia tsutsugamushi*), and lice as vectors for epidemic typhus (*R. prowazekii*) (*1*).

In Chile, the last outbreak of epidemic typhus began in 1933 and continued through 1939 (*2*). In the following years, effective control and sanitary measures were developed and implemented. No new cases of rickettsial disease have been reported in this country since 1976 (*3*). Scrub typhus, caused by *O. tsutsugamushi*, which is usually transmitted by trombiculid mites in Asia, northern Australia, and the western Pacific region has never been described in Chile (*4*). Although sporadic cases of scrub typhus have been reported well outside the traditionally endemic regions (*5**,**6*), no reports are known of scrub typhus being acquired in the Western Hemisphere (*4*). In addition, no human case of rickettsial spotted fever has been documented in Chile, although there is evidence of rickettsial infections in dogs (*7*) and of the presence of *R. felis* in cats and cat fleas (*8*). We report a case of scrub typhus–like illness in Chile.

## Materials and Methods

The patient was a previously healthy 54-year-old man who recalled having been bitten by terrestrial leeches on several occasions but not by ticks. He was hospitalized after symptoms including a high-grade fever developed. During treatment, a black eschar with an erythematous halo on the left leg was found. A biopsy sample from the leg eschar was submitted to the laboratory for histopathologic analysis and subjected to microscopy. A routine blood chemistry panel was analyzed. In addition, an ELISA to detect *O. tsutsugamushi*–specific immunoglobulin G was performed with acute-phase and convalescent-phase serum samples (*9*).

The same skin biopsy samples of the eschar and rash were submitted for molecular biology analysis. DNA was extracted from the skin biopsy samples by using the QIAamp Tissue Kit (QIAGEN, Hilden, Germany) according to the manufacturer’s instructions. The prokaryotic 16S rRNA gene was amplified and sequenced by using described primers (*10*). The PCR products were purified with the GFX DNA gel band purification kit (GE Healthcare, Piscataway, NJ, USA) and sequenced by using the BigDye Terminator version 3.1 Cycle Sequencing Kit and a 310 Genetic Analyzer (Applied Biosystems, Foster City, CA, USA).

Sequences obtained from the leg and arm samples were assembled by using the Sequencher DNA Software (Gene Codes Corporation, Ann Arbor, MI, USA) and were judged to be identical. The sequences were initially compared with other 16S rRNA sequences in GenBank by using the National Center for Biotechnology Information BLAST network service software (*11*). The sequence of the biopsy sample (referred to as the Chiloé Island sample) has been deposited in GenBank under accession no. HM155110. Sequence differences between the Chiloé Island sample and isolates of *O. tsutsugamushi* were determined after aligning 16S rRNA sequences, using ClustalX in MEGA4 (*12*).

The patient had been involved in ecological studies at a university field camp at the southern end of Chiloé Island, in southern Chile. The region is rainy and has abundant natural vegetation and evergreen forests. During January 2006, the patient spent 3 weeks on a field study (sleeping in a log cabin) with daily forest incursions. He recalled having been bitten by terrestrial leeches on several occasions but not by ticks.

**Figure 1:**
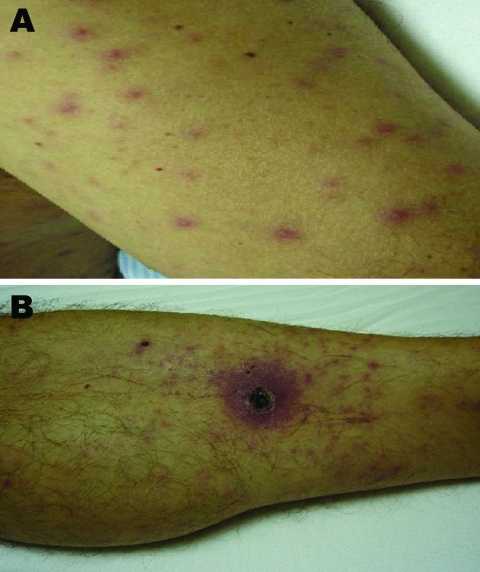
Evidence of acute infection of the skin and subcutaneous tissue in patient admitted for treatment of scrub typhus-like symptoms in Chile. A) Rash on admission, left arm. B) Necrotizing eschar with erythematous halo over the left leg.

A week after returning to the capital, Santiago, and 6 days before his admission to the hospital, a high-grade fever, headache, myalgias, and scanty dry cough developed. Four days later, a rash appeared in the abdominal region that progressed to his face and limbs. At admission, the patient had an axillary temperature of 39ºC, pulse 101 beats/min, blood pressure 110/75 mm Hg, and bilateral conjunctival suffusion. He had an extensive rash on his face, trunk, and limbs but not on the palms and soles, with a microvesicular center in some of the lesions (Figure 1, panel A). A black eschar with an erythematous halo on the left leg was found. He recalled having been bitten by a leech ≈3 weeks before (Figure 1, panel B).

## Results

**Figure 2:**
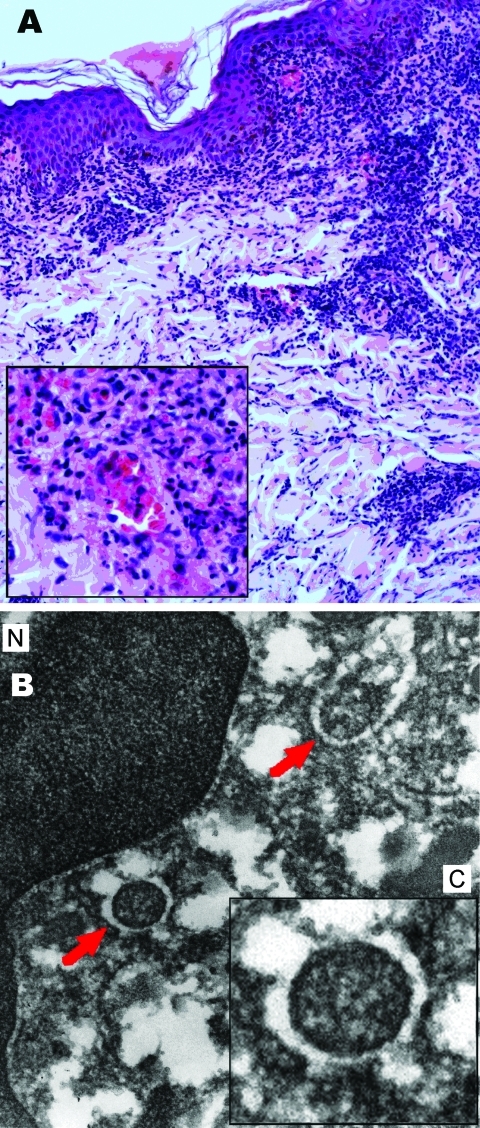
Results of biopsy analysis of tissue sample from eschar on the left leg of patient admitted for treatment of scrub typhus–like symptoms, Chile. A) Leukocytoclastic vasculitis. Hematoxylin and eosin stained; original magnification ×200, inset ×400. B) Endothelial cell, showing nucleus (N) within the cytoplasm (C, inset). Arrows show similar round and oval organisms, electron-dense, surrounded by electron-lucent halo of rickettsial type microorganisms. Electron microscopy; original magnification ×15,000, inset ×20,000.

Dermis and subcutaneous fat showed a necrotizing leukocytoclastic vasculitis, perivascular infiltrates with lymphocytes and macrophages, and extravasation of erythrocytes (Figure 2, panel A). Gram, Giemsa, and Warthin-Starry silver stains did not show any microorganisms. A tissue sample recovered from a paraffin-embedded sample for electron microscopy, showed round and oval rickettsia-like microorganisms, maximum diameter 0.2–0.5 μm, inside the cytoplasm endothelial cells (Figure 2, panel B).

Blood tests showed a leukocyte count of 9,200 cells/mm^3^, with 28% immature forms and a slight elevation of hepatic aminotransferase levels (aspartate aminotransferase 198 U/L [reference range 10–40 U/L], alanine aminotransferase 256 U/L [reference range 10–55 U/L], and alkaline phosphatase 338 U/L [reference range 45–115 U/L]) with normal bilirubin level. Blood cultures (2 sets) were negative as were serologic test results for measles, varicella, leptospirosis, and HIV. A spotted fever rickettsiosis was suspected and doxycycline (100 mg 2×/d) was started on the day after admission. ELISA to detect *O. tsutsugamushi* specific immunoglobulin G on the acute-phase sample had no reactivity (titer <100) to the Karp, Kato, Gilliam combined whole-cell ELISA antigen, whereas the convalescent-phase serum sample, collected 4 months after hospitalization, had a titer of 400, showing that seroconversion had occurred between the 2 time points. These same serum samples were examined for reactivity to ELISA antigens from rickettsial spotted fever group and rickettsial typhus group. The serum samples were nonreactive to both groups of antigens (titer <100). Skin punch biopsy specimens (4 mm) from the skin lesions (eschar and rash) were taken on the second day after admission. After the second day of antimicrobial drug therapy, the patient’s general condition markedly improved and the fever subsided. On the following days the skin lesions began to fade. The patient was discharged in good health condition on the fifth day of hospitalization.

DNA from the biopsy sample was used for a molecular analysis in order to identify the infectious agent. Analysis found that the sequence of the 16S rRNA gene obtained from the skin biopsy specimen showed ≈97% sequence similarity with isolates of *O. tsutsugamushi* (39–44 nt differences in a 1,265-bp alignment of the 16S rRNA gene). Recently, a case of scrub typhus was reported from Dubai outside the normal range of the disease (*5*). A similar level of difference (42-nt differences) was seen when the Chiloé Island sample was compared with the *O.* *chuto* sp. nov. sample from Dubai.

Sequence differences among various isolates of *O. tsutsugamushi* ranged from 1 to18 nt, and *O.* *chuto* sp. nov. differed from the *O. tsutsugamushi* samples by 23 to 31 nt differences. Comparisons were also made with 16S rRNA sequences from 2 leech-associated forms placed within the genus *Rickettsia* (*13**,**14*), and with taxa from genus *Neorickettsia*, a group of obligate intracellular forms placed within the Ehrlichiaceae that have been isolated from trematodes. When compared with 2 isolates that represent presumptive members of the genus *Rickettsia* that are endosymbionts of leeches in Japan, sequence similarity to the Chiloé Island sample averaged 91.9% (97-nt differences). As expected, if no special selective effect resulted from being associated with leeches as a vector, the degree of sequence change is equivalent to that found when the 16S rRNA gene sequence of the Chiloé Island sample is compared with other members of *Rickettsia* not associated with leeches (sequence similarity 91.6%).

Isolates of *O. tsutsugamushi* averaged 91.8% sequence similarity to other members of *Rickettsia*, and the *O.* *chuto* sp. nov. sample averaged 92.2% similarity to non-leech *Rickettsia* spp. The 16S rRNA gene sequence showed even greater divergence when the Chiloé Island sample was compared with 3 *Neorickettsia* spp., with sequence similarity averaging 83.4% (average diversity 215 nt). Isolates of *O. tsutsugamushi* averaged 82.7% sequence similarity to *Neorickettsia*, and the *O.* *chuto* sp. nov. sample averaged 82.2% similarity. Taken together, these results are consistent with 16S rRNA gene sequence of the Chiloé Island sample being representative of an *Orientia* spp.–like form.

**Figure 3:**
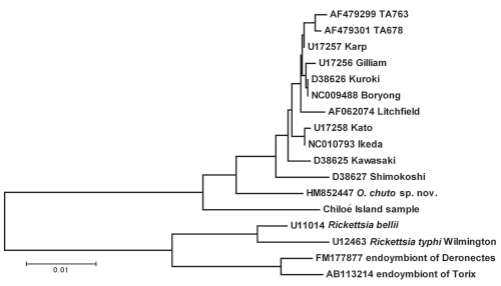
Evolutionary relationships of Chiloé Island sample compared with other isolates of *Orientia tsutsugamushi,* with *O.* *chuto* sp. nov. and with taxa from *Rickettsia*, determined by the method of neighbor joining (*15*). The tree is drawn to scale; scale bar indicates nucleotide substitutions per site. Numbers on branches represent percentage of 1,000 bootstrap replicates that include the enclosed clade. Entries on the tree are identified by GenBank accession number and isolate name.

The phylogenetic relationship of the Chiloé Island sample with other isolates of *O. tsutsugamushi* was inferred by using the neighbor-joining method (*15*), and is shown in Figure 3. The evolutionary distances were computed using the maximum composite–likelihood method (*16*) and are in units of the number of base substitutions per site. All positions containing gaps and missing data were eliminated from the dataset (complete deletion option). A total of 1,256 positions were identified in the final dataset. Phylogenetic analyses were conducted in MEGA4 (*12*). Sequences of the 16S rRNA gene sequences from the 3 taxa of *Neorickettsia* were used to root the tree. The Chiloé Island sample is well differentiated from sequences from *O. tsutsugamushi* and *O.* *chuto* sp. nov., being separated in 100% of bootstrap replicates of the analysis, but clearly significantly closer to the samples classified within *Orientia*, compared with other rickettsiae in the sequence databases.

Two other PCR assays were performed that were specific for sequences of *O. tsutsugamushi* and the genus *Rickettsia* by using primers that targeted a portion of the *groEL* gene because of its higher power of differentiation between closely related taxa. However, no appropriate size amplicons were produced. Moreover, results of a quantitative real-time PCR assay for *O. tsutsugamushi* (*17*) and 2 PCR assays (47 kDa/*HtrA* gene and *groEL* gene) used with the DNA preparation extracted from acute-phase serum sample (2 days after admission, 1 day after antimicrobial drug treatment) were negative (data not shown).

In the study of the new form, *O.* *chuto* sp. nov., from Dubai, the 47-kDa/*HtrA* gene was amplified. The degree of sequence divergence compared with isolates of *O. tsutsugamushi* was substantial, averaging >17% (*5*). Given that the Chiloé Island sample shows almost twice as much divergence from *O. tsutsugamushi* for the 16S rRNA sequence compared with *O.* *chuto* sp. nov., it is not unreasonable that substitutions in the PCR primer sites of the Chilean sample exist, explaining the negative results that were found in our study.

## Discussion

We describe a case of rickettsiosis acquired in Chiloé Island, where the local population is mostly of the Huilliche ethnic background. One of the ancient local legends refers to a disease developing in persons who penetrate the jungle, with the development of high fever and red spots all over the body. However, no scientific medical report had confirmed this finding.

Even though the existence of scrub typhus has never been recorded in Chile, its vector, the trombiculid mite (Acari: Trombiculidae), has been recently described in wetlands from a distant region of southern Chile, although not on Chiloé Island (*18*). Our patient recalled specifically having been bitten by a leech in the site where an eschar later developed. Terrestrial leeches are common on Chiloé Island vegetation. These include members mainly from the family *Mesobdellidae*, including the species Mesobdella *gematta* and *Nesophilaemon skottsbergi* (*19*). The leeches live among trees, ferns, bushes, and fallen leaves. All are sanguivorous parasites of vertebrate animals, and local persons are frequently exposed to leech bites on the island. Rickettsiae have been reported in leeches in Japan (*13**,**14*). In those studies, the glossiphoniid leech species harbored bacteria of the genus *Rickettsia*, as assessed by electron microscopy and PCR analysis.

The results of analysis of the 16S rRNA gene sequence suggest that the sample reported represents a previously unreported, divergent form (species) of *Orientia* spp.–like bacteria. The degree of sequence differentiation from isolates of *Orientia* spp. previously studied in Asia and the Middle East indicates that the Chiloé Island sample is not simply a transplanted form from Asia that happened to be discovered in Chile, but rather it represents a long divergent lineage and may be indicative that other *Orientia* spp.–like pathogens are to be found outside southern and eastern Asia or northern Australia. The difficulty of obtaining PCR amplification of additional sequences, such as the GroEL and 47-kD protein genes, would be consistent with the identification of a new lineage divergent from Asian forms of *O. tsutsugamushi*. Moreover, the reactivities of the serum samples to *O. tsutsugamushi* Karp, Kato, Gilliam ELISA antigens (titer 400) suggest that the cross-reactivity of assay antigens to those of the new sample may exist but be limited, again consistent with a divergent lineage.

Future steps following the case presented will involve investigating whether Chiloé Island’s leeches carry rickettsiae and whether these rickettsiae, according to additional DNA sequence analysis, are closely related to members of *O. tsutsugamushi* or if they represent a new lineage within or closely related to the known forms of *Orientia*. If no related rickettsiae are identified from leeches, an alternative possibility is that trombiculid mites are present on Chiloé Island and that these are the vectors of the pathogen. However, the observation that the eschar developed at the site of leech attachment would appear to argue against an alternative vector. Nevertheless, chiggers, the proven vector free-living stage in the mite life cycle that feeds on the vertebrate hosts, are small and easily overlooked. Thus, a mite cannot be positively excluded as the vector in this case. Whether other sporadic cases of human rickettsial illness may have occurred in that area should also be the subject of future investigation.
